# George Fu Gao: the past and future of Ebola

**DOI:** 10.1093/nsr/nwv010

**Published:** 2015-02-22

**Authors:** Mu-ming Poo, Ling Wang

**Affiliations:** Mu-ming Poo is the Director of CAS Center for Excellence in Brain Science and Executive Associate Editor of NSR, and Ling Wang is a science news reporter based in Beijing

## Abstract

Newly returned from the frontline of the battle against Ebola in West Africa, Dr George Fu Gao talked to NSR on this largest Ebola epidemic in history. As Deputy Director-General of the Chinese Center for Disease Control and Prevention (China CDC), an Academician of the Chinese Academy of Sciences and a fellow of the Third World Academy of Sciences (TWAS), Dr Gao has been personally involved in the fight against acute infectious diseases both on the laboratory bench and in the field for many years. Recalling the sight of an Ebola-infected young man stumbling to his death on the street and observing the continuing climb of patient statistics, he anxiously calls for larger global help to affected countries. The help provided by the world community to West Africa was ‘too late and insufficient’, and in this age of globalization ‘no country can care only for herself alone’. In this interview, Dr Gao also reviews the history, progression, and prognosis of the Ebola epidemic, the cause of failure in the early prevention and containment of the disease, the role of government policies and public health systems, and global prospects and strategies for similar acute infectious diseases in the future.

## PERSISTING CRISIS

**NSR**: Having returned to China, are you still closely monitoring Ebola? What is your assessment of the current situation?

**Gao**: Certainly. The China CDC is providing a daily report on Ebola, and I am closely watching the situation in West Africa. We cannot be too optimistic about the current status. Among the three most affected countries, Liberia appears to have passed the peak, although the decline is rather slow and fluctuates; Sierra Leone is currently lingering near the peak, showing an apparent plateau; Guinea's situation fluctuates greatly, with signs of a major return of Ebola. Based on the overall trend, the best scenario is that the epidemic may be curtailed by the coming June, but some scientists estimate that this is more likely to occur by the end of year 2015.

**NSR**: Ebola virus was discovered in 1976, but has never caused the large-scale infection we face today, why?

**Gao:** The most important features in preventing acute infectious diseases are part of the ‘Four Early’ strategy: early discovery, early diagnosis, early quarantine, and early intervention. In this recent epidemic, there was failure in all four of these strategies. The main problem lies in the management: the three West African countries did not pay much attention to this matter in the beginning, and it became hard to contain the disease once it spread.

Ebola began in December 2013, with the first patient being a two-year-old boy in Guinea. When the Pasteur Institute confirmed the cause to be Ebola virus in March 2014, Ebola had already spread to Liberia. Sierra Leone claimed to have attended to this matter but did not install an effective measure. By May 2014, the disease had already been spreading for half a year and entered into Sierra Leone. If sufficient attention were given at that time, Ebola would not have become so unmanageable later. After May 2014, as the situation became more serious, the inability of the three affected countries to deal with the epidemic was apparent, and other countries did not react quickly enough.

**NSR**: As reported in the media, many countries and organizations deployed help to West Africa. Did this help to curtail the disease?

**Figure 1:**
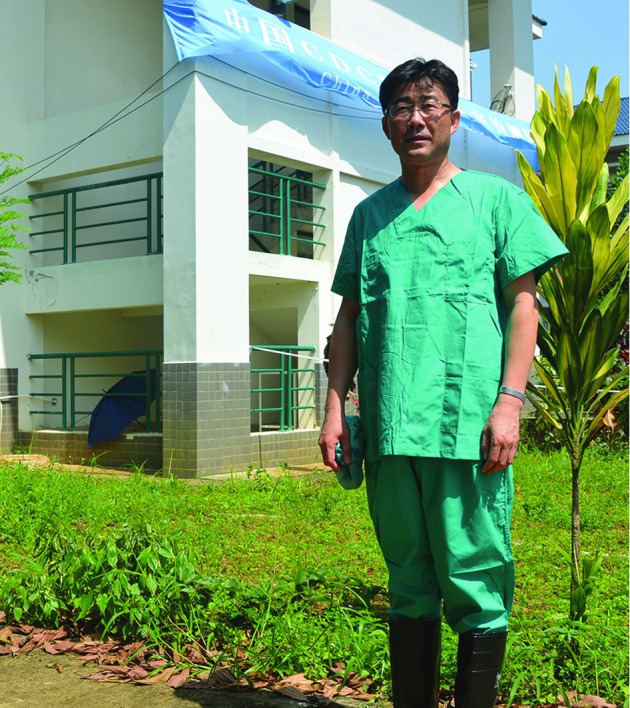
Dr George Fu Gao, Deputy Director-General of China CDC, fighting against Ebola in Sierra Leone *(Courtesy of China CDC)*

The main problem lies in the management: the three West African countries did not pay much attention to this matter in the beginning, and it became hard to contain the disease once it spread.

—George Fu Gao

**Gao**: There was some impact, but I think the help was too late and not strong enough. As I have just said, the three countries themselves were no longer capable of dealing with the disease by May 2014. The public health facilities are poor in these countries—there are no diagnostic capabilities, not enough healthcare workers, and insufficient medicine and basic nutrition or food for sustaining the patients. The fact is that the World Health Organization (WHO) did not openly announce the seriousness of the epidemic until August 2014. Even then, most countries only reacted at the level of discussion—for setting rules and operation procedures, without really reaching the frontline to curtail the disease. To fight Ebola in countries like Sierra Leone, ‘teach them how to fish’ is not sufficient; you must also ‘give them the fish’. There is an immediate need for trained healthcare workers on the ground, helping the diagnosis, quarantine, and intervention of the disease.

Many people have said that death rate for Ebola is anywhere between 20% and 90%. This is neither a rigorous nor correct statement. If I said the rate for an event to occur is between 0 and 100%, you would say that I was talking nonsense. With regard to Ebola, if there is intervention the death rate is 20%, and the rate will reach 90% if there is no intervention. During the early phase of the epidemic, some Ebola patients did not even receive a saline infusion (to maintain electrolyte balance and nutrition), and the death rate was high. Now, with vein infusions, the death rate is dropping, even in the absence of an antiviral treatment. In other words, Ebola is not so horrific; its lethality can be controlled with proper actions.

## CRIPPLING POLICIES AND THE PUBLIC HEALTH SYSTEM

**NSR**: Was the slow reaction to the Ebola epidemic related to the systems and policies of local governments?

**Gao**: Indeed. As a former British colony, Sierra Leone's policies on biomedical ethics followed closely those in western countries. This aspect is acutely reflected in matters related to Ebola. For example, a Japanese-developed anti-influenza virus inhibitor, Avigan or Favipiravir, has been shown to be effective against Ebola in animal models, but the clinical trials for such a drug in Sierra Leone is hampered by many factors, including ethics. Even when the drug was available, there were many meetings to deal with the issues of distribution of the drug, who should get the drug etc., while the situation continued to worsen.

Furthermore, in China, there is a multisector coordination system (including all the relevant ministries under the State Council, led by the National Health and Family Planning Commission, NHFPC) related to acute infectious diseases, and compulsory quarantine can be quickly implemented when suspected cases are found. This cannot be done in Sierra Leone as there is no supporting regulation. There is also the lack of hospitals and facilities for accepting the patients. Furthermore, healthcare workers strike from time to time due to the lack of proper personal protection or delayed wage payments. These conditions are hard to imagine in China.

**NSR**: In other words, if Ebola spreads to China, we will be able to deal with it.

**Gao**: Yes. China now has a well-established system for responding to the spread of infectious diseases, unless the infected individuals go to hide in remote areas, an unlikely scenario. Thus, we do not need to worry, as long as we can react early. Of course, there are still many unanswered questions regarding Ebola virus and its infection. We must take every step seriously.

**NSR**: Vaccine is important for the fight against pathogens. Why is a vaccine still unavailable so many years after the discovery of Ebola virus?

**Gao**: An Ebola vaccine is obviously a matter of concern for public health, but pharmaceutical companies are driven by profit and are responsible to their shareholders. They will not actively develop the vaccine until the government decides to invest on it. We all saw the consequence of their slow reaction.

**NSR**: What is the current status of vaccine development?

**Gao**: There are three vaccines currently in clinical trials. One is being developed by GlaxoSmithKline, based on chimpanzee adenovirus (AdV) 3 and Ebola glycoprotein; the second is being developed by NewLink Genetics and Merck Vaccine, based on attenuated vesicular stomatitis virus and the glycoprotein; and the third one is being developed by Dr Wei Chen of the Chinese Academy of Military Medical Sciences, based on human AdV5 and the glycoprotein. There is a problem using AdV5; 80% of people have antibodies against it, and whether this will affect clinical effectiveness remains unknown. Other vaccines are also under various stages of development.

**NSR**: Given their clinical trial status, these vaccines cannot be used widely at present. Is that right?

**Gao**: No. This is a scientific as well as a management issue. I have frequently said, in the course of history, biological safety and ethics were ignored, but it seems to be overemphasized at present. It has been a long delay for the above-mentioned vaccines to be set for clinical trials during the outbreak because of the existing ethical and management hurdles. In China, the Ebola vaccine, developed by Dr Wei Chen, could be used in human trials prior to animal tests, though it is controversial. During the SARS epidemic, we considered taking blood samples from SARS patients to develop ‘therapeutic plasma’. This simply could not be done in Sierra Leone for the Ebola convalescent antisera. The same is true for sequencing of the virus. This has to be done in the USA and requires approval from Sierra Leone.

**Figure 2:**
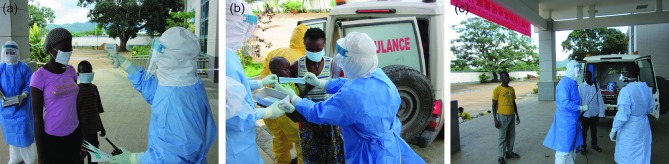
(a) Chinese medical workers placing masks on patients who are being monitored for potential infection *(By courtesy of the 302 Military Hospital of China)*. (b) A Chinese nurse distributing masks to the people with suspected Ebola infection and taking their temperature *(By courtesy of the 302 Military Hospital of China)*. (c) A transport ambulance upon its arrival at the hospital undergoing disinfection *(By courtesy of the China Medical Tribune)*.

## LEARNING FROM HISTORY

**NSR**: Through viral sequencing, a Harvard team found in June 2014 that Ebola virus has mutated with a rate doubling that found over the past 40 years, why?

**Gao**: As the virus spreads in the human population, it continues to mutate and adapt to new human hosts. In the past, the infection normally spread for only 2–3 months, but this time the spread lasted for more than six months by June 2014, allowing more time for the mutated virus to survive. Now another half a year has passed, we would like to know the extent of further mutation beyond that reported earlier. This is why I suggested that our NHFPC should coordinate the shipment of a BGI's sequencer to West Africa for on-site sequencing.

**NSR**: Does rapid mutation of Ebola virus make it more dangerous?

**Gao**: Some people worried that Ebola virus may convert into a ‘influenza-like virus’ in terms of rapid mutations (antigenic drift) (requiring different vaccines for different subtypes). As you know, we need to be vaccinated every year to be protected against the seasonal flu. If Ebola virus mutates as quickly/frequently as influenza virus, the vaccine needs to be continuously renewed. We need to have a vaccination for influenza each year because each vaccine can only target one virus subtype or virus strain. A vaccine developed now for Ebola may become obsolete by the time it is used for vaccination. For example, the H1N1 and H3N2 vaccines are currently being used to prevent influenza, but the vaccine suitable for the mutated virus next season needs to be developed. Such vaccine development requires large investment and has a very high risk. Thus, research and development of Ebola vaccine belongs to the realm of public health. It must be a government-led effort, and little initiative can be expected from the pharmaceutical industry.

**NSR**: To some extent, didn't the Ebola epidemic repeat the pathway of the AIDS epidemic?

**Gao**: In the beginning, both Ebola and AIDS could be considered to be diseases of the poor. Both originated from Africa where medical care and living conditions are poor, and both were neglected by the world in the beginning. When AIDS reached Europe in the 1980s and began to spread by sexual

encounter, it caught the world's attention. For 40 years, Ebola was active only in local regions in Africa. Without the present epidemic, it probably would not cause a ripple in the media.

**NSR**: Can you comment on the AIDS situation in China?

**Gao**: AIDS is still very serious in China. The situation has become more serious than that in the USA and Europe. The pathway for AIDS transmission in China is now clear. During the early period of post-cultural revolution, poverty was widespread in China and many poor people relied on selling blood plasma to make a living. After separating the plasma, the blood cells were infused back to the donor. Since the blood was mixed among many people before re-infusion, the virus carried by one donor quickly spread to others. Most AIDS cases in Henan and Shanxi villages were caused by this blood infusion practice.

When people became more affluent in the 1990s, drug addiction became the main pathway of infection, and high incidence rates were concentrated in Yunnan, Guangxi, and Xinjiang. In this century, with increasing social diversification and tolerance, (both homo- and hetero-) sexual transmission became the main pathway of propagation. In particular, incidence rate due to male homosexual transmission has risen rapidly in recent years, and college students have become a high-risk incidence population. This indicates that we have much to do in social transparency and education, including distribution of free condoms in public places and education against dangerous sexual behaviors.

**NSR**: How is the current status and effectiveness of AIDS therapy?

If Ebola virus mutates as quickly/frequently as influenza virus, the vaccine needs to be continuously renewed. A vaccine developed now for Ebola may become obsolete by the time it is used for vaccination.

—George Fu Gao

**Gao**: The current therapy is rather effective. The drug is pushing gradually towards the cure of AIDS. However, the present approach in developing an AIDS vaccine is not very hopeful. I think the AIDS vaccine research has reached a dead end. Just following the successful example of polio vaccine—using a vaccine to activate immune responses—may not be the best approach. When a virus has already weakened the immune system, is it reasonable to further force the immune system to fight the virus? We need a new conceptual scheme and new approaches.

**NSR**: With regard to influenza, is it possible to develop a universal vaccine against all subtypes of a virus?

**Gao**: I have studied influenza virus for a long time. Personally, I think it is not possible to develop a universal vaccine. We can now find universal neutralizing antibodies (to influenza virus) in human blood, but when the virus really invades humans, these antibodies seem to be in hibernation, showing no protective reaction. We do not understand the underlying mechanism yet. We need a new theoretical framework to guide the search for a universal vaccine.

**NSR**: What do you think is the most urgent measure needed for controlling the Ebola epidemic in West Africa?

**Gao**: I think China has accumulated much useful experience in dealing with acute infectious diseases. With minor modifications, the Chinese practices could be adapted to West Africa. In particular, supportive therapy for patients is more urgent at present than preventive approaches such as vaccine development.

## VIRAL INFECTION MECHANISMS

**NSR**: What do we know about the origin of Ebola?

**Gao**: Scientists now believe that Ebola virus originates from bats, some of which are distributed along the belt above the equator, from Sierra Leone, Republic of Congo, to the Philippines and our regions of Yunnan and Hong Kong. It is not clear whether these bats migrate along the equator-like migratory birds. If so, then it can spread the virus to different parts of the world without human carriers.

**NSR**: Doesn't SARS virus also originate from bats?

**Gao**: Indeed. With regard to the origin of SARS virus, Zhengli Shi's team at Wuhan Institute of Virology of CAS were the first to report, in the journal *Nature*, the isolation of a strain of virus from horseshoe bats (named ‘WIV1’) that is very similar to SARS coronavirus (SARS-CoV). They also showed that WIV1 binds to the SARS-CoV receptor, thus providing direct evidence that bat-to-human transmission is plausible. K. Y. Yuen of Hong Kong University has also discovered many viruses in bats (named HKU1, HKU2…) and we, in collaboration with K. Y. Yuen showed that HKU4 can use the receptor for the newly emerged Middle East Respiratory Syndrome coronavirus to enter human cells, although the efficiency is low. This represents another example of bat-originated respiratory syndrome viruses. In a paper published in 2013 in *Nature*, my team at the Institute of Microbiology of CAS reported the molecular structural mechanism by which MERS virus interacts with the human cell surface receptor (CD26). Such structural information is very useful for designing both antiviral drugs and prophylactic vaccines.

**NSR**: How does a virus invade human cells? Does it always need a surface receptor?

**Gao**: The great majority of viruses need to bind to specific cell surface receptors before entering the cell. The SARS receptor is called ACE2 and MERS receptor is CD26. There are indeed viruses that may not require receptors for invading the cell. For example, the receptor for Dengue fever and other rather ancient viruses (e.g. yellow fever virus and West Nile virus) has never been found. As Ebola virus can infect multiple somatic cell types, it also may not require a specific cell surface receptor, but this remains to be determined. An unusual feature of Ebola virus is the prolonged incubation period—as long as 21 days. This is rather rare among acute infectious diseases. Male Ebola patients can carry virus in their semen for three months during the recovery period. It is not clear why.

**NSR**: Does this mean the potential for the sexual transmission of Ebola?

**Gao**: There is no evidence for sexual transmission yet. Human eyes and testicles are immune privileged organs that could tolerate an invading virus without eliciting inflammatory immune responses and thus should not be infected. We are currently studying why Ebola virus can reach the semen—it means that the testicles are infected. The paradox of the immune privilege and virus infection-resistance is not fully understood for these organs.

**NSR**: What are the remaining mysteries in Ebola virus?

**Gao**: I think the mechanism of cross-species transmission needs to be understood. Many newly emerged infectious diseases involve infection in both humans and animals, but some viruses affect humans but not animals, why? Understanding whether and how a virus affecting one species become infectious in another species is critical in developing effective preventive measures. Our understanding of molecular mechanisms via research on influenza virus over the years greatly helped the analysis of the viral infection mechanism when avian flu epidemic appeared.

We have recently established a CAS Center for Influenza Research and Early-warning. One of our goals is to solve the problem of cross-species transmission through multidisciplinary approaches. Regarding virus propagation by migratory birds, for example, zoologists may examine the migratory behaviors of birds, virologists and molecular biologists can study the mechanism of infection of avian viruses on human cells, and China CDC, which has established a national network of infectious diseases monitoring networks throughout China since the SARS pandemic, can provide immediate information on disease incidence in various local regions. Integrating multiple resources of information will allow us to understand the nature of the virus, and whether and how it spread cross species.

**NSR**: Some scientists propose to use ‘gain of function’ (GOF) to predict potential threats of viral mutation on human populations. What is your view on this approach?

**Gao**: Frankly, I am not in favor of the GOF approach, although there are pro and con arguments. My point is that the process of gene mutation and natural selection involves many more combinatorial scenarios than the human mind can imagine, and how could one select among all possibilities and provide comprehensive tests? I think a more direct and effective approach is to strengthen the capability of monitoring disease-causing viruses and finding solutions to curtail their spread. For example, we have monitored H5- and H9-type influenza viruses over a long period; when similar infectious diseases appear, we can rely upon existing methods and platforms to analyze the virus and propose a solution. During the H7N9 avian flu epidemic in 2013, we were able to identify the problem and

Many newly emerged infectious diseases involve infection in both humans and animals, but some viruses affect humans but not animals. Understanding whether and how a virus affecting one species become infectious in another species is critical in developing effective preventive measures.

—George Fu Gao

suggest the closing of markets for live poultry trading, a measure that prevented further spread of the flu.

**NSR**: Ultimately, the capability in the prevention and control of acute infectious diseases testifies the social and economic strength of the country, doesn't it?

**Gao**: Indeed, acute infectious diseases could be regarded not only as a medical problem but also a social problem. It could evoke social unrest and become a political problem. The SARS epidemic resulted in the dismissal of the minister of health in China; Sierra Leone has replaced their minister twice since the Ebola outbreak. It is also a diplomatic problem as many countries are involved in dealing with Ebola. As a poor country to begin with, Sierra Leone received another devastating blow by Ebola. The only consolation may be some improvement of its public health system after this epidemic.

Finally, this epidemic testifies to the fact that infectious diseases are a global issue that requires collaborative efforts from many countries. Given the absence of adequate public health systems in many developing countries, affecting their ability to deal with acute infectious diseases, more developed countries must provide rapid and effective responses when such an epidemic occurs again in the future.

